# Cardiovascular magnetic resonance imaging of cardiac remodeling and strain analysis in adolescent obesity

**DOI:** 10.1186/1532-429X-18-S1-P284

**Published:** 2016-01-27

**Authors:** Anna Schmidt, Alessandro Satriano, Kate Fenwick, Madeline Arnold, Andrew G Howarth, Nowell M Fine, Daniele Pacaud, Todd Anderson, Bobby Heydari, James A White, Matthias G Friedrich

**Affiliations:** 1 Stephenson Cardiovascular Magnetic Resonance Imaging Centre, Libin Cardiovascular Institute, Calgary, AB Canada; 2 grid.22072.350000000419367697Department of Paediatrics, University of Calgary, Calgary, AB Canada; 3 grid.22072.350000000419367697Department of Medicine, Division of Cardiology, University of Calgary, Calgary, AB Canada; 4 grid.14709.3b0000000419368649Division of Cardiology, Department of Medicine, McGill University, Montreal, QC Canada

## Background

The increasing worldwide prevalence of childhood obesity portends the development of type 2 diabetes and cardiovascular disease (CVD) in this population. Cardiovascular Magnetic Resonance (CMR) imaging provides a sensitive imaging tool that may afford early detection of structural and functional alterations secondary to obesity. This pilot study explores the potential for CMR imaging, inclusive of left ventricular (LV) volume and mass analysis, Native T1 mapping and 4D strain analysis to identify such changes related to obesity in adolescence.

## Methods

Eleven subjects between 10 and 20 years were prospectively recruited from a Pediatric Weight clinic, and defined as obese if they were ≥95^th^ percentile of weight for their age. Fourteen age and gender-matched healthy weight control subjects were also enrolled. All subjects had baseline anthropometric and laboratory measures in addition to a standardized CMR imaging protocol, inclusive of routine multi-planar cine imaging and native T1 Mapping using the shMOLLI pulse sequence. Left Ventricular (LV) volumetric analysis and T1 mapping was blindly analyzed using cvi42 (Circle Cardiovascular Inc, Calgary). 4D strain analysis was performed using GIUSEPPE in-house software.

## Results

The mean age of the obese group was 14 ± 1 years versus 14 ± 2 years in controls (p=0.572). BMI values were higher in the obese cohort (32 ± 6 and 21 ± 4 kg/m^2^, p < 0.0001) as was systolic blood pressure (SBP), serum lipid levels and markers of insulin resistance. LV ejection fraction was non-significantly elevated in the obese group (62.8 ± 4.7% vs 59.1 ± 5.4%, p = 0.084). A trend was seen in elevation of LV Mass index (26.1 ± 5.9 vs 21.6 ± 6.7 g/m^2.7^, p = 0.084) with mean segmental wall thickness being significantly elevated in the obese cohort (5.5 ± 0.8 vs 4.6 ± 0.8 mm, p = 0.019). BMI was positively correlated with LV Mass Index (r = 0.648, p < 0.0001), wall thickness (r = 0.708, p < 0.0001) and mass:volume ratio (r = 0.415, p = 0.039). 4D strain analysis revealed significant elevations in peak circumferential endocardial strain (-20.5 ± 2.1 vs -18.7 ± 2.0% p = 0.04) and peak longitudinal endocardial strain (-16.7 ± 1.6 vs -14.4 ± 1.9% p = 0.006) in the obese cohort, consistent with a hyper-contractile state. Circumferential epicardial strain was not significantly different between groups, however, was significantly positively correlated with systolic blood pressure (r = 0.472, p = 0.023) and LV Mass Index (r = 0.484, p = 0.019). Native T1 mapping was not significantly different between the groups (954 ± 21 vs 954 ± 31 ms, p = 0.985).

## Conclusions

In this pilot study we identify pediatric obesity to be associated with objective elevations in LV mass, wall thickness and Mass:Volume ratio. In this "early stage" of remodeling these structural changes were not associated with elevation in native T1, a surrogate marker of tissue fibrosis. Strain analysis revealed a hyper-contractile state, likely representing a combined contribution of elevated systolic blood pressure with compensatory myocyte hypertrophy.

